# Assessment of primary care doctor’s diagnosis of difficult-to-treat asthma in school children

**DOI:** 10.1186/2045-7022-3-S1-O11

**Published:** 2013-05-03

**Authors:** Ole D Wolthers

**Affiliations:** 1 Asthma and Allergy Clinic, Denmark

## Rationale

In primary care settings difficult-to-treat asthma may be interpreted as severe asthma. Little is known about diagnostic outcomes in children referred to secondary pediatric referral centers with an established primary care doctor’s diagnosis of difficult-to-treat bronchial asthma.

## Objective

To assess diagnostic outcome in school children referred to a secondary pediatric referral centre with an established primary care doctor’s diagnosis of difficult-to-treat bronchial asthma.

## Methods

482 consecutively referred children aged 5-14 (mean 7.9) years, 99 girls (21%) and 383 boys (79%) with a primary care doctor’s referral diagnosis of difficult-to-treat asthma were included from the prospective Asthma in a Secondary Pediatric Referral Centre Study (ASP 2002) in the present survey. At referral and during a 6 months evaluation period patient characteristics, history, symptoms, signs and results of type 1 allergy tests, spirometry, post bronchial beta-2 agonist dilation tests, 4-weeks daily measurement of peak flow rates, corticosteroid reversibility trials and exercise challenge tests were entered into a pre-defined electronic form. The secondary referral centre (SRC) diagnosis of asthma was based on these data.

## Results

A diagnosis of asthma was confirmed in 200 (41%), whereas it could not be confirmed in 282 (59%) of the children. Allergic rhinoconjunctivitis was diagnosed in 96 (48%) in the confirmed group, in 87 (31%) in the not confirmed group. A variety of differential diagnoses was made in the children in whom asthma was not confirmed.

## Conclusions

In more than half of school age children with a primary care doctor’s diagnosis of difficult-to-treat asthma referred to a secondary pediatric referral centre the diagnosis may not be confirmed. Sensitivity and specificity of the diagnosis of asthma in school children made in primary care settings need further improvement.

